# The Effect of Non-Invasive Brain Stimulation on the Downregulation of Negative Emotions: A Meta-Analysis

**DOI:** 10.3390/brainsci12060786

**Published:** 2022-06-15

**Authors:** Qingqing Zhang, Xiaoming Li, Xinying Liu, Shanshan Liu, Mengzhu Zhang, Yueling Liu, Chunyan Zhu, Kai Wang

**Affiliations:** 1 The School of Mental Health and Psychological Sciences, Anhui Medical University, Hefei 230032, China; qing9812142021@163.com (Q.Z.); psyxiaoming@126.com (X.L.); L15665315060@163.com (X.L.); liushanshan010903@163.com (S.L.); zhangmengzhu@163.com (M.Z.); Lucuma@163.com (Y.L.); wangkai1964@126.com (K.W.); 2 Anhui Province Key Laboratory of Cognition and Neuropsychiatric Disorders, Hefei 230032, China; 3 Collaborative Innovation Center of Neuropsychiatric Disorders and Mental Health, Hefei 230032, China; 4 Institute of Artificial Intelligence, Hefei Comprehensive National Science Center, Hefei 230011, China; 5 Department of Neurology, The First Affiliated Hospital of Anhui Medical University, Hefei 230022, China

**Keywords:** non-invasive brain stimulation, TMS, tDCS, emotion regulation, meta-analysis

## Abstract

(1) Background: Emotion regulation (ER) is regarded as a core treatment target for depression and other mental illnesses. In recent years, non-invasive brain stimulation (NIBS) has been extensively used as an intervention for mental illnesses, but there has been no systematic review conducted regarding its effect on emotion regulation. Therefore, we conducted a meta-analysis of the effectiveness of NIBS for emotion regulation; (2) Methods: Systematic searches were conducted in Embase, Web of Science, PubMed, and Cochrane Library. We analyzed the effects of NIBS on tasks assessing emotion regulation using a random-effects model, and further explored the moderating role of the following factors on transcranial direct current stimulation (tDCS) studies by conducting subgroup analyses and meta-regression: target electrode placement, return electrode placement, current intensity, target electrode size, and duration of intervention; (3) Results: A total of 17 studies were included. Our meta-analysis indicated a small but significant effect of NIBS on the downregulation of negative emotions. Separate analyses indicated that repetitive transcranial magnetic stimulation (rTMS) had a medium and significant effect on the downregulation of negative emotions, whereas tDCS had no significant effect. Subgroup analyses showed that the effect of tDCS was moderated by target and return electrode placemen; (4) Conclusions: These results indicate that NIBS had a positive effect on the downregulation of negative emotions. The stimulation protocols should be carefully considered and the underlying mechanisms should be further explored.

## 1. Introduction

Emotion regulation (ER) encompasses a complex set of extrinsic and intrinsic control processes by which an individual attempts to evaluate, modify, and monitor the occurrence, duration, and intensity of emotional reactions, thus, influencing emotions [1,2]. According to forms of the regulation goal, emotion regulation can be divided into explicit/conscious emotion regulation and implicit/nonconscious emotion regulation. Explicit emotion regulation goals involve a conscious desire to up- or down-regulate one’s emotions (e.g., to increase pleasure or reduce sadness), while implicit emotion regulation goals change emotional reactions independent of consciousness [3]. negative emotions arise from to terrible events, in which people make efforts to regulate their negative emotions to maintain psychological well-being. Numerous studies corroborated the relevance of the abnormalities of regulating negative emotions and mental disorders, and the abnormalities were also increasingly regarded as underlying the etiology and maintenance of mental disorders [4,5]. Except for symptoms such as anhedonia, loss of energy and interest, insomnia and concentration problems, emotion dysregulation is also one of the key symptoms of depression [6,7]. This dysregulation could explain the positive emotion deficits in depression [8]. Anxiety disorder and borderline personality disorder display similar patterns to depression in terms of emotion regulation strategies. Findings show anxiety disorder and borderline personality disorder are positively associated with rumination and avoidance [9,10], suggesting similar patterns to depression in terms of emotion regulation strategies. An event-related potential (ERP) study also confirmed that people with schizophrenia have some difficulty regulating negative emotions as they are ineffective at implementing reappraisal and distraction [11]. In addition, it is well documented that children with autism spectrum disorder often demonstrate difficulty in emotional regulation [12,13]. Therefore, emotion regulation has become the focus of attention in the treatment of depression, anxiety, autism spectrum disorder and other illnesses [12,14]. At present, emotion dysregulation is mainly intervened through drug therapy [15] and an array of contemporary psychotherapies, such as cognitive-behavioral therapy [16], emotion regulation therapy [17], and dialectical behavioral therapy [18]. Medication and psychotherapy have limitations such as drug side effects and high cost. Thus, more economical, convenient, non-invasive and highly acceptable treatment is worth further exploring [19].

In recent years, non-invasive brain stimulation (NIBS) such as transcranial magnetic stimulation (TMS) and transcranial direct current stimulation (tDCS) have been widely used in clinical research. The tDCS induces a neuron membrane polarization by delivering a weak electric direct current to the scalp [20], rTMS modulates brain activity by delivering strong magnetic pulses through electromagnetic coils placed over the scalp [21], and both can potentially induce long-term changes in brain activity in a safe and tolerable manner. They have also been shown to be effective as an intervention for posttraumatic stress disorder, schizophrenia, obsessive-compulsive disorder, and depression [22,23,24,25,26]. Neuroimaging studies suggested that the cognitive impairment observed in these illnesses was related to abnormal activity in particular brain regions [27,28,29,30], and NIBS could affect neural excitability to improve cognitive function [31,32], including attention and working memory [33]. A meta-analysis also evaluated the effect of TMS and tDCS on cognitive functioning in patients with brain disorders, such as dementia, Parkinson’s disease, stroke, and traumatic brain injury, revealed that both TMS and tDCS elicit a small trans-diagnostic effect on working memory [34]. For healthy individuals, excitatory NIBS has a positive effect on executive functioning [35]. Inhibitory control is also improved after tDCS in single-session studies [36]. Moreover, tDCS can help to reduce stress-related emotional reactivity [37].

Since emotion regulation is considered as key treatment target for many mental diseases, such as borderline personality disorder and depression [7,10], extensive studies have also been carried out to examine the relationship between NIBS and emotion regulation, especially in downregulation of negative emotions [38,39]. Emotion regulation was mainly measured by two methods in the studies using NIBS to affect emotion regulation: self-report emotion regulation questionnaire and experimentally instructed emotion regulation task. The former focused on the differences in the frequency of use of emotion regulation strategies measured by questionnaire before and after tDCS or TMS interventions, to assess the effect of intervention on emotion regulation [40,41,42]. The latter directly observed the regulating effect of NIBS on the up- and down-regulation of emotions by stimulating related brain regions during or before the implementation of the emotion regulation task, and the interaction between stimulation and emotion regulation strategies can also be observed at the same time [43,44,45]. In these studies, participants report their emotional states after a particular emotion regulation strategy during or after stimulation. Researchers assess the change in emotion to indicate the effects of the stimulation and strategy on emotion [46]. Therefore, we focused on the studies that assessed the effects of NIBS on experimentally instructed emotion regulation processes in this study.

In general, previous studies of the effect of TMS and tDCS on emotion regulation task have involved two types of research objectives. The first objective was to clarify the causal neural mechanisms underlying emotion regulation using NIBS to alter neural activity in the stimulated regions. For instance, a study employed TMS to elucidate the special causal relationship between the dorsolateral prefrontal cortex (DLPFC)/ventrolateral prefrontal cortex (VLPFC) and explicit downregulation of negative emotions, and found that the VLPFC and DLPFC displayed a relative functional specificity for reappraisal and distraction strategies [47]. Other studies used single-pulse TMS and continuous theta burst stimulation (cTBS) and also found that the left VLPFC regulates negative emotions with functional specificity and the left temporoparietal junction (TPJ) contributed to distancing [48,49]. Another objective was to directly test the effect of NIBS on the emotion regulation of special individual and different emotional stimuli, for instance, to explore whether tDCS could improve downregulation of emotions of social exclusion in depression-prone individuals [50]. In some studies, clinical samples such as patients with alcohol use disorder (AUD) and obsessive-compulsive disorder (OCD) were also compared with healthy individuals to observe their emotional downregulation using functional magnetic resonance imaging and rTMS [51,52]. Although many of these studies have explored the relationship between NIBS and emotion regulation, especially in downregulation of negative emotions, there has been no review of the literature conducted to date.

Despite a good level of evidence for the efficacy of NIBS on emotion regulation, there is no consensus on it and it is still controversial. Several studies suggested that NIBS had a positive effect on the downregulation of negative emotions [47,48,53]. However, some empirical studies showed that the efficacy was limited [49,52], for example, a study found that unilateral stimulation of moderate intensity over left PFC may not be sufficient to elicit therapeutic effects for depression [54]. Therefore, it is necessary to adopt a meta-analysis to quantify the effectiveness of NIBS on emotion regulation and identify the factors that influence the outcome of the intervention. In view of the current studies, we mainly analyzed the effect of NIBS on the downregulation of negative emotions to evaluate the effect of intervention. Moreover, emotion regulation was generally operationalized by two conditions of behavioral task, the “regulate” condition and the “look” condition, thus we further evaluated the effects of NIBS on the maintenance of negative emotions by meta-analysis. In addition, a study with the emotion regulation task found that when tDCS was applied in the DLPFC it did not regulate emotional reactions, but when applied in the VLPFC it had a good effect [55]. So, we conducted subgroup analyses and meta-regression to explore the moderators.

## 2. Materials and Methods

A systematic literature search was performed in line with the guidelines of PRISMA [56,57]. The protocol of the study was registered on PROSPERO in advance (https://www.crd.york.ac.uk/prospero/) and the registration number is CRD42021272188.

### 2.1. Information Sources and Search Strategy

In this study, the databases Embase, Web of Science, PubMed, and Cochrane Library were comprehensively searched. We mainly searched for articles about the effects of TMS and tDCS on emotion regulation. We retrieved articles up to 10 March 2022. The following key concept combinations were used for the scoping search: [NIBS] AND [emotion regulation]. The key concept “NIBS” includes the following terms: “noninvasive brain stimulation”, “transcranial magnetic stimulation”, “theta burst stimulation”, “transcranial direct current stimulation”, or “transcranial electrical stimulation”. The key concept “emotion regulation” included the following terms: “emotion regulation”, “reappraisal”, “suppression”, “distraction”, “rumination”, “acceptance”, or “avoidance”. An elaborate description of the search terms can be found in the Supplementary Material.

### 2.2. Literature Review

**Figure 1:**
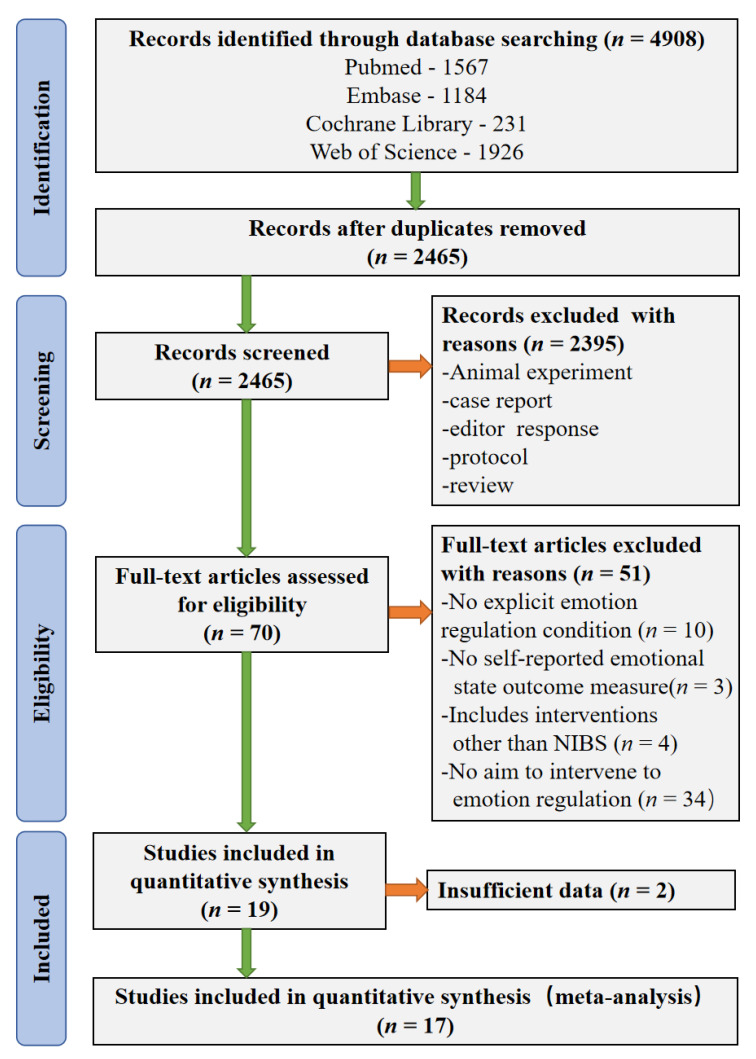
PRISMA flow diagram of literature search and study selection.

The studies were independently screened on the eligibility criteria by two authors (Q. Z. and X. L.). The missing data were acquired by contacting authors. The discrepancies between two reviewers were resolved by judgement with another reviewer (S. L.). The details of the literature search strategy are presented in Figure 1.

### 2.3. Eligibility Criteria

Articles were included if they met the following criteria: (a) The article was published in English and peer reviewed. (b) The aim of article was to test the effect of NIBS (TMS or tDCS) on the downregulation of negative emotions. (c) The participants included healthy volunteers or clinical populations, aged 18–60 years. (d) An eligible control condition restricted to sham stimulation was included in the study design. (e) The study procedure included the downregulating condition of emotions. The downregulating condition was induced in an attempt to enhance negative emotion downregulation to images shown by reappraising the content or other emotion regulation strategies. (f) The article reports the data of a subjective negative emotional state after emotion regulation induction measured within the time frame of NIBS (after) effects. This involved self-report measures of experienced negative emotions after emotion regulation induction (i.e., emotional ratings). (h) In the study, only NIBS was used as an experimental intervention. 

### 2.4. Data Extraction 

The authors Q. Z. and M. Z. independently extracted the following data from the included articles: the basic information of the study (authors, years, journal, and experimental type); the demographics of the participants in studies (gender, sample size, age, and sample type); the emotion regulation task details (task name, the emotional stimuli, emotion regulation strategy); the details of stimulation (stimulation regions, technique, parameters, timing, and the type of sham stimulation); the main result (the means and standard deviations (SDs) of the self-reported negative emotional ratings in the emotion regulation condition in the active and sham stimulation group).

If the required data were missing, the following steps were taken. GetData Graph Digitizer (version 2.25) was used to extract the means and SDs that were only found in the figures. If the study conducted multiple trial types (e.g., social pain pictures versus individual pain pictures), these outcome measures were combined by recalculating the mean and the pooled SD. If relevant data were not reported, the Supplementary Material of the study was searched for the data. If relevant data were still not available, we contacted the corresponding authors to provide the data. We excluded studies where we sent five email reminders to the corresponding authors without receiving a response or the authors were unable to provide data on time for some reason. In addition, in most studies, higher emotional ratings corresponded to stronger negative emotions. If a study used an inverse rating, the mean was transformed by subtracting the original mean scores from the maximum rating.

### 2.5. Study Quality and Risk of Bias Assessment

The methodological quality of included studies was assessed according to the Physical Therapy Evidence Database (PEDro) scoring system [58]. The checklist has 11 items that were answered with yes (score = 1) or no (score = 0). The first item was not used in calculating the final score. The discrepancies between the individual raters were resolved based on discussions. Furthermore, funnel plots were produced to assess risk of publication bias, and the asymmetry of funnel plots was tested by an Egger’s regression test.

### 2.6. Data Analysis

To evaluate the effect of NIBS on the downregulation of negative emotions, we focused on outcomes of self-reported negative emotional ratings in the condition of downregulating. The specific method was to compare the difference of negative emotional ratings between the active and sham stimulation groups, and the difference reflected the effect of NIBS on the downregulation of negative emotions. In addition, to reflect the unique role of NIBS on the downregulation of negative emotions, we further evaluated the impact of NIBS on the maintenance of negative emotions. We focused on outcomes of self-reported negative emotional ratings in the condition of maintenance. The method was to compare the difference of negative emotional ratings between the active and sham stimulation groups, and the difference reflected the effect of NIBS on the maintenance of negative emotions.

All quantitative analyses were carried out in R (version 4.0.4) package Metafor [59]. The standardized mean differences (SMD) and the Hedge’s g [60] were conducted to observe the effects of NIBS on the downregulation of negative emotions and the maintenance of negative emotions. Here, negative values indicated better downregulation of negative emotions after active stimulation. Some studies measured more than one effect size, and this nesting of effects within studies violates assumptions of independence and may contribute to potential bias in the results. To adjust for the correlation of effects within studies, a multi-level model analysis was conducted using the rma.mv function of the “Metafor” R package [61,62]. Random-effects models [63] were implemented to assess the effect of NIBS. We used *Q* and I^2^ statistics to estimate the magnitude of heterogeneity [64].

Furthermore, the effect of rTMS differed with the pulse frequency; for example, high-frequency rTMS (HF-rTMS) reduced the self-reported experience of negative emotions through the increased cortical excitability associated with emotion regulation, while low-frequency rTMS and continuous theta burst stimulation increased negative emotional reactions through reducing cortical excitability [49,65,66,67]. Therefore, we first conducted a meta-analysis for all effect sizes to assess the effect of NIBS, and further performed a meta-analysis in healthy people of HF-rTMS and tDCS respectively. Finally, as tDCS analysis revealed substantial heterogeneity, we analyzed the moderating role of the following factors on tDCS intervention in the downregulation of negative emotions: target electrode placement, return electrode placement, current intensity, target electrode size, and duration of intervention. The categorical moderators were conducted by subgroup analysis [68]. For continuous moderators, we used a restricted maximum likelihood model with the Knapp-Hartung method to conduct meta-regression analyses.

## 3. Results

### 3.1. Search Results

As shown in Figure 1, the systematic literature search yielded 4908 studies. The titles and abstracts of 2465 studies were assessed for eligibility after removing duplicate research. The full text evaluation was carried out in the 70 potentially relevant studies. Of these, 19 studies fulfilled the eligibility criteria. Amongst them, we did not receive a response from the corresponding author about the missing data in one study [43], and the authors of other study were unable to provide data until three months later due to isolation [69]. Finally, 17 studies were included in the meta-analysis [47,48,49,50,51,52,53,54,55,70,71,72,73,74,75,76,77]. Each of included studies had the condition of downregulate negative emotions in study procedure, but two of them did not have the condition of maintenance of negative emotions [53,77].

### 3.2. Study Characteristics

The 17 studies included a total of 1253 participants, of which 136 received both active and sham stimulation, 609 only received active stimulation, and 508 only received sham stimulation. In addition, 150 of the participants had a psychiatric disorder, including 39 with AUD, 39 with depression, 33 with internet gaming disorder, and 39 with OCD, while the other participants were healthy. Four studies used HF-rTMS [47,51,52,70], of which one study used LF-rTMS also [52], one study used single-pulse TMS [48], one study used cTBS [49], and 11 studies used tDCS [50,53,54,55,71,72,73,74,75,76,77]. Materials from the above studies included pictures or emotional stimulus which only induced negative emotions. Amongst them, including specific negative emotions such as social pain [47,50,70,71], fear [52], disgust [72] and negative emotional memories [77]. Other study characteristics can be found in Table 1 and Table 2.

### 3.3. Meta-Analysis

#### 3.3.1. Overall Meta-Analysis

**Figure 2:**
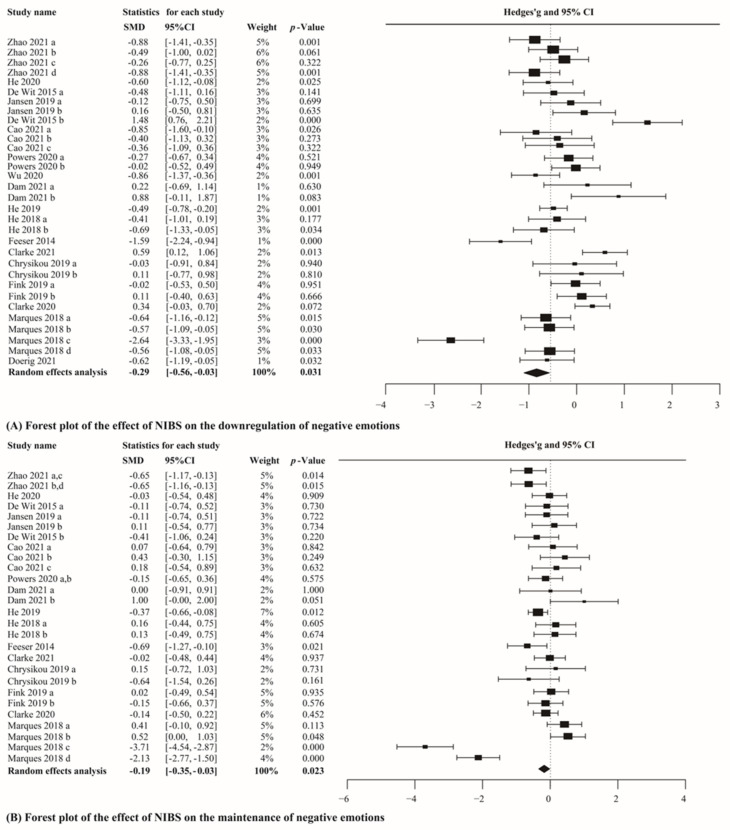
Results of the meta-analyses for overall NIBS studies. (a–d) Different effect sizes from the same study [47,48,49,50,51,52,53,54,55,70,71,72,73,74,75,76,77].

We firstly analyzed the data in the downregulating condition of 17 studies, including 1253 participants. The results showed a significant difference in the self-reported negative emotion between the active and sham stimulation groups in the downregulating condition (g = −0.29, CI_95%_ = [−0.56, −0.03], Z-value = −2.153, *p* = 0.031, Figure 2A). While we analyzed the data in the maintaining negative emotions condition of 15 studies, including 1170 participants. The results showed a small but significant difference in the self-reported negative emotion between the active and sham stimulation groups in the maintaining condition (g = −0.19, CI_95%_ = [−0.35, −0.03], Z-value = −2.274, *p* = 0.023, Figure 2B). In addition, the interpretation of these results was restricted because the analysis revealed substantial heterogeneity (*Q* = 148.43, *p* < 0.001, I^2^ = 79.11%; *Q* = 143.77, I^2^ = 80.50%, *p* < 0.001). Due to the large heterogeneity of the analysis, further meta-analyses were conducted independently in healthy people according to the stimulus types.

#### 3.3.2. rTMS

**Figure 3:**
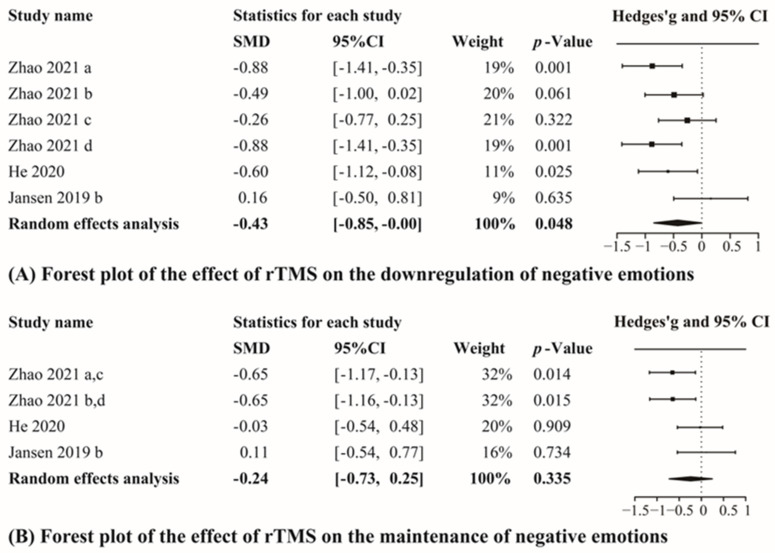
Results of the meta-analyses for rTMS studies. (a–d) Different effect sizes from the same study [47,51,70].

We separately analyzed the data of four studies in the downregulating condition and the maintaining condition, including 185 healthy participants. Of these, 109 only received active stimulation, and 76 only received sham stimulation. The results of the analysis in downregulating condition showed no significant heterogeneity (*Q* = 8.79, *p* = 0.118, I^2^ = 43.15%) and that self-reported emotion significantly differed between active and sham stimulation groups (g = −0.43, CI_95%_ = [−0.85, −0.00], Z-value = −1.98, *p* = 0.048, Figure 3A). The results of the analysis in maintaining condition also showed no significant heterogeneity (*Q* = 5.97, *p* = 0.113, I^2^ = 35.05%), but we found no significant difference between active and sham stimulation (g = −0.24, CI_95%_ = [−0.73, 0.25], Z-value = −0.96, *p* = 0.335, Figure 3B). 

#### 3.3.3. tDCS

**Figure 4:**
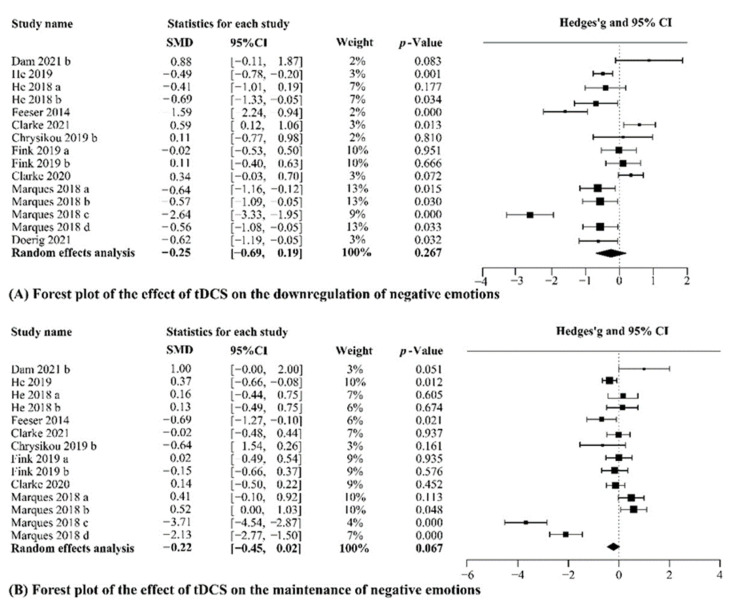
Results of the meta-analyses for tDCS studies. (a–d) Different effect sizes from the same study [50,54,55,71,72,73,74,75,76,77].

We firstly analyzed the data in the downregulating condition of 10 studies, including 897 healthy participants. Of these, 58 received both active and sham stimulation, 423 only received active stimulation, and 416 only received sham stimulation. The meta-analysis showed significant heterogeneity (*Q* = 102.18, *p* < 0.001, I^2^ = 86.30%), and we did not observe any difference in the self-reported emotion between active and sham stimulation groups in the downregulating condition (g = −0.25, CI_95%_ = [−0.69, 0.19], Z-value = −1.11, *p* = 0.267, Figure 4A). While we analyzed the data in the maintaining negative emotions condition of 9 studies, including healthy 847 participants. Of these, 58 received both active and sham stimulation, 398 only received active stimulation, and 391 only received sham stimulation. The meta-analysis showed significant heterogeneity (*Q* = 130.21, *p* < 0.001, I^2^ = 90.02%), and we also did not observe any difference in the self-reported emotion between active and sham stimulation groups in the maintaining condition (g = −0.22, CI_95%_ = [−0.45, 0.02], Z-value = −1.83, *p* = 0.067, Figure 4B). Considering large heterogeneity revealed by meta-analyses of the tDCS studies in healthy people, we further investigated moderator variables to follow up on heterogeneity.

### 3.4. Evaluation of Moderators

#### 3.4.1. Subgroup Analysis

We conducted subgroup analyses for some a priori selected categorical moderators (target electrode placement and return electrode placement) in studies of tDCS intervention in emotion downregulation in healthy individuals. The results revealed the target electrode placement and return electrode placement were significant moderating variables (*p* = 0.000; *p* = 0.019). Table 3 shows the results for the individual studies varying in either target or return electrode position.

#### 3.4.2. Meta-Regression Analysis

We conducted meta-regression analyses for continuous moderators (current intensity, target electrode size, duration of intervention). The analysis showed current intensity (*β* = 0.00), target electrode size (*β* = −0.05) and the duration of intervention (*β* = −0.04) were not significantly related to the effect of tDCS on emotion downregulation. Detailed results are shown in Table 4.

### 3.5. Quality and Risk of Bias

**Figure 5:**
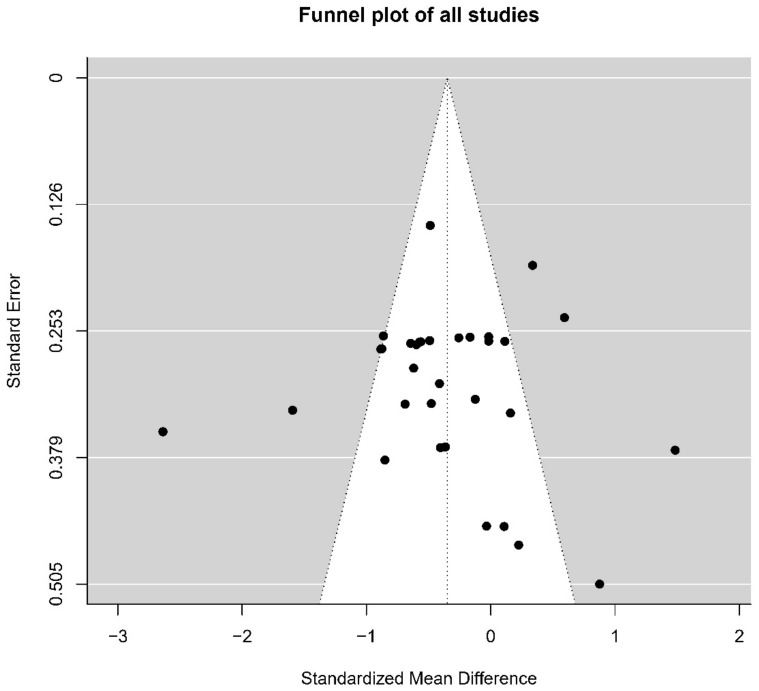
Funnel plot of studies on NIBS intervention on the downregulation negative of emotions.

No publication biases were suggested by visual examination of funnel plots for all studies. The Egger’s regression test showed the absence of asymmetry (*p* = 0.473, Figure 5). PEDro scores were assessed and calculated for each study (see Supplementary Material). The mean score of all studies was 7.88, the SD was 1.11. Literature quality assessment found that most of the studies had incomplete methodological reports, such as blinding procedures that were not explained. In addition, although many studies randomly grouped participants, they did not report how the random sequence was generated and whether group assignments were hidden from the researchers.

## 4. Discussion

This meta-analysis investigated the effects of NIBS on the downregulation of negative emotions and the maintenance of negative emotions in single-session designs with healthy or clinical populations. Overall, we documented a small and significant excitatory effect of NIBS on the downregulation of negative emotions (g = −0.29), and a significant effect on the maintenance of negative emotions (g = −0.19). Further analyses showed that rTMS had a medium and significant effect on the downregulation of negative emotions in healthy populations (g = −0.43), but had no significant effect on the maintenance of negative emotions, while tDCS also had no significant effect on the downregulation and maintenance of negative emotions in healthy populations.

Although the results of a previous meta-analysis showed a single session of NIBS was insufficient to reduce negative emotional reactivity [37], our results differed. The contribution of NIBS to the downregulation of negative emotions was confirmed in our study. The regulation of emotions involves a well-established bilateral network of prefrontal control regions including parietal and temporal regions as well as the DLPFC, VLPFC, and dorsomedial prefrontal cortex (DMPFC) [78,79]. NIBS could impact the level of cortical excitability of the DLPFC/VLPFC by modulating synaptic plasticity or increasing the firing rate of neurons to promote better emotion downregulation through a functionally interactive network of cortical-limbic pathways [80,81]. To be specific, NIBS may inhibit the amygdala by activating prefrontal regions (e.g., DLPFC), thereby reducing emotional reactivity towards negative emotional content contributes to downregulation [74,82,83]. Previous behavioral studies also indicated rTMS decreased stress hormonal responses and heart rate variability after a stress-inducing task [84,85]. In addition, NIBS could result in diminished attentional engagement to negative stimuli to help downregulate emotions [86]; stimulating the lateral prefrontal cortex indirectly affected related areas that play a role in attention (e.g., anterior cingulate cortex) [87]. Direct stimulation of the dorsal prefrontal cortical region affects cognitive control of emotions, which is a core feature of the emotion regulation strategies used [88]. Interestingly, we also concluded that the effect of rTMS was better than tDCS on the downregulation of negative emotions; this has not been previously investigated. The navigation system of rTMS aided precise stimulation, and the electric field strength induced by rTMS was more intense than tDCS, which may account for this result [89]. In addition, rTMS regulates attention better than tDCS [32], thus a good emotional downregulation effect might be achieved. Besides, the insignificant intervention effect of tDCS suggested that better stimulation protocols and more precise targets are required to modulate the emotion regulation networks to improve negative emotions.

Furthermore, we documented a small and significant positive effect of NIBS on the maintenance of negative emotions, that is, NIBS plays an excitatory role in reducing negative emotional responses. This indicates that NIBS may not significantly promote the use of explicit emotion regulation strategies, but promote the downregulation of negative emotions by reducing negative emotional responses. In fact, few behavioral studies documented a positive effect of NIBS on the emotion regulation strategies [51,72,74,75,76]. Neuroimaging studies of NIBS also indicated that the increased DLPFC activity was independent of emotion regulation strategies [52,54]. These results suggested the existence of both explicit and implicit emotion regulation in experimentally instructed emotion regulation tasks [3]. NIBS did not promote the regulating advantage of explicit strategies, but promoted implicit regulation, which led to the downregulation of emotions [90]. However, the interpretation of this result is very limited because the analysis showed large heterogeneity and the results of separate analyses of rTMS and tDCS did not reach statistical significance. With respect to the rTMS and tDCS effects without significant results, it also could be considered that the number of studies on rTMS and tDCS is small, thus any effect of stimulation on the maintenance of negative emotions may be potentially obscured. In addition, a single session of rTMS or tDCS may not be enough to cause a significant effect on negative emotional responses [37]. Therefore, further studies into augmenting the efficacy of NIBS protocols on emotion regulation is valuable.

Moreover, the potential sources of heterogeneity in tDCS studies were investigated across subgroup and meta-regression analyses. Our subgroup analyses found the effective size of the emotion downregulation of tDCS targeting the rDLPFC (g = −0.69) and the rVLPFC (g = −0.45) was significantly larger than that of tDCS targeting the lDLPFC (g = −0.11). The result provided a reference for selecting which brain regions to target for intervention in regulating negative emotions, the rDLPFC and rVLPFC should be considered. Previous studies suggested that the neural mechanisms of emotion regulation include DLPFC and VLPFC [3]. For example, in the process of emotion regulation, holding in mind regulatory goals and controlling the focus of attention on strategy relevant information depends on dlPFC-parietal control network [91]; the function of the VLPFC involves the selection of goal-appropriate interpretations and inhibition of inappropriate ones, which is essential to reappraisal [3]. In addition, The VLPFC plays a special role in the selective attention of emotional stimuli and can better modulate amygdala activity through the rostral anterior cingulate cortex (rACC), which is part of the ventral emotion processing system [92,93]. Moreover, hemispheric asymmetry in negative emotion regulation has been observed. The intervention on right DLPFC resulted in a better effect than on the left, which may be due to the fact that negative emotional processing as occurring predominantly in the right hemisphere [94]. Meanwhile, we found that the effect of different return electrode placements should be considered regarding the effect of tDCS on the downregulation of negative emotions. Ultimately, of course, tDCS works by combining both electrodes, especially in bilateral electrode settings [36]. Although there were no significant results in the meta-regression analysis, the results of the above subgroup analyses provided us with ideas to explain the sources of heterogeneity in tDCS studies but must be interpreted with extreme caution since only a few studies were included in the subgroup analysis. Thus, further studies are required.

Our meta-analysis had several limitations. First, emotion regulation was measured by subjective self-report, which may be biased by reporting and therefore not a sensitive measure of regulatory performance. In the future, further studies need to apply a more precise measure of emotion regulation to evaluate the effect sizes for NIBS. Second, some outcome categories and subgroup analyses included a small number of studies, leading to uncertainty about the effect size of the results. Future research could address these concerns with larger samples. Third, the literature included in our analysis has certain quality risks. Thus, the results of these analyses were limited by the high heterogeneity present, which may result from the different experimental designs and stimuli. Thus, we hope that more high-quality literature can be included in future studies. In addition, a potential limitation of our meta-analysis is that we could not exhaustively explore all possible moderators and the classification of moderators is not detailed enough (e.g., electrode placement).

## 5. Conclusions

To conclude, the results of these analyses largely supported our primary hypothesis, that is, a single session of NIBS had a positive effect on emotion regulation, especially in the downregulation of negative emotions. Therefore, NIBS may help alleviate the emotion dysregulation symptoms of mental and neurological disorders, NIBS should be considered more widely in clinical treatment. Since the small number of studies with clinical populations included, our study is insufficient to effectively analyze the effects of NIBS on emotion regulation of different diseases. In future studies, researchers should aim to assess the effect of NIBS on emotion regulation in different mental and neurological disorders, to better assess whether NIBS has a diagnostic effect on emotion regulation. Meanwhile, intervention methods to improve the use of emotion regulation strategies may need to be further explored, as a single session of rTMS or tDCS was not found to have a stable positive effect on it in this study. In addition, this study found that the effect of the intervention is still affected by many factors, such as electrode placement. In the future, we need further studies to clarify stimulation parameters, optimal stimulation site, intervention time, and the number of sessions for NIBS on improving emotion regulation, in order to find the most effective, convenient and tolerable intervention protocols. 

## Figures and Tables

**Table 1:** Characteristics of included transcranial magnetic stimulation studies.

	Reference	Design, Sample Type, Size *n*(Active)|*n*(Control)	Age (M ± SD or Range), Sex Ratio (M/F)	Coil Position (Localization Method)	Control Condition	Stimulation Frequency, Quantity	Protocol	Experiment Stimuli	Task (Emotion Regulation Strategy)	Outcome Measure
HF- rTMS	Zhao et al. 2021 [47] a	Between-subject, Healthy, 30|30	19.8 ± 1.6, (15/15) ^1^	Right VLPFC (F8)	Control site stimulation (vertex, Cz)	10 Hz, 624 pulses	Offline	Social exclusion pictures	ERT (Reappraisal)	Perceived negative emotion in picture (Likert: 1–9)
	Zhao et al. 2021 [47] b	Between-subject, Healthy, 30|30	19.2 ± 1.4, (15/15) ^1^	Right DLPFC (F4)	Control site stimulation (vertex, Cz)	10 Hz, 624 pulses	Offline	Social exclusion pictures	ERT (Reappraisal)	Perceived negative emotion in picture (Likert: 1–9)
	Zhao et al. 2021 [47] c	Between-subject, Healthy, 30|30	19.8 ± 1.6, (15/15) ^1^	Right VLPFC (F8)	Control site stimulation (vertex, Cz)	10 Hz, 624 pulses	Offline	Social exclusion pictures	ERT (Distraction)	Perceived negative emotion in picture (Likert: 1–9)
	Zhao et al. 2021 [47] d	Between-subject, Healthy, 30|30	19.2 ± 1.4, (15/15) ^1^	Right DLPFC (F4)	Control site stimulation (vertex, Cz)	10 Hz, 624 pulses	Offline	Social exclusion pictures	ERT (Distraction)	Perceived negative emotion in picture (Likert: 1–9)
	He et al. 2020 [70]	Between-subject, Healthy, 30|29	21.3 ± 1.8, (17/13) ^1^	Right VLPFC (F8)	Coil tilted at 90°	10 Hz, 1170 pulses	Offline	Social exclusion pictures	ERT (Reappraisal)	Perceived negative emotion in picture (Likert: 1–9)
	De Wit et al. 2015 [52] a	Between-subject, OCD, 19|20	38.4 ± 10.0, (21/22) ^2^	Left DLPFC (Individual)	Control site stimulation (vertex)	10 Hz, 3000 pulses	Offline	Fearful IAPS pictures	ERT (Reappraisal)	Distress (Likert: 1–100)
	Jansen et al. 2019 [51] a	Between-subject, AUD, 19|20	41.64 ± 8.63, (26/13) ^2^	Right DLPFC (Individual)	Coil tilted at 90°	10 Hz, 3000 pulses	Offline	Negative IAPS images	ERT (Reappraisal)	Negative emotional experience (VAS: 0–100)
	Jansen et al. 2019 [51] b	Between-subject, Healthy, 19|17	43.75 ± 10.90, (20/16) ^2^	Right DLPFC (Individual)	Coil tilted at 90°	10 Hz, 3000 pulses	Offline	Negative IAPS images	ERT (Reappraisal)	Negative emotional experience (VAS: 0–100)
single-pulse TMS	Cao et al. 2021 [48] a	Within-subject, Healthy, 15|~	23.53 ± 4.44, (9/6) ^1^	Left VLPFC (between AF7 and F7)	Control site stimulation (vertex, Cz)	single pulse, at 300 ms after picture stimuli onset	Online	Negative IAPS images	CRT (Reappraisal)	Negative emotional experience (Likert: 1–9)
	Cao et al. 2021 [48] b	Within-subject, Healthy, 15|~	23.53 ± 4.44, (9/6) ^1^	Left VLPFC (between AF7 and F7)	Control site stimulation (vertex, Cz)	single pulse, at 300 ms after picture stimuli onset	Online	Negative IAPS images	CRT (Reappraisal)	Negative emotional experience (Likert: 1–9)
	Cao et al. 2021 [48] c	Within-subject, Healthy, 15|~	23.53 ± 4.44, (9/6) ^1^	Left VLPFC (between AF7 and F7)	Control site stimulation (vertex, Cz)	double pulse, at 300\3300 ms after picture stimuli onset	Online	Negative IAPS images	CRT (Reappraisal)	Negative emotional experience (Likert: 1–9)
LF- rTMS	De Wit et al. 2015 [52] b	Between-subject, Healthy, 19|18	39.6 ± 11.4, (18/20) ^2^	Left DLPFC (Individual)	Control site stimulation (vertex)	1 Hz, 3000 pulses	Offline	Fearful IAPS pictures	ERT (Reappraisal)	Distress (Likert: 1–100)
cTBS	Powers et al. 2020 [49] a	Within-subject, Healthy, 30|~	18–39, ~ ^2^	TPJ (Individual)	Sham coil	bursts of 3 pulses at 50 Hz delivered at a rate of 5 Hz, 300 pulses	Offline	Negative IAPS images	ODT (Objective distancing)	Negative emotional experience (Likert: 1–7)
	Powers et al. 2020 [49] b	Within-subject, Healthy,30|~	18–39, ~ ^2^	TPJ (Individual)	Sham coil	bursts of 3 pulses at 50 Hz delivered at a rate of 5 Hz, 300 pulses	Offline	Negative IAPS images	ODT (Distraction)	Negative emotional experience (Likert: 1–7)

^1^ The demographical characteristics of the active group; ^2^ The demographical characteristics of the active and control groups; ~ The included studies did not explicitly report sex ratios or equal numbers of active and control groups in the within-subject study; a–d: Different effect sizes from the same study; AUD = alcohol use disorder; CRT = cognitive reappraisal task; cTBS = continuous theta burst stimulation; DLPFC = dorsolateral prefrontal cortex; ERT = emotion regulation task; HF-rTMS = high-frequency repetitive transcranial magnetic stimulation; IAPS = international affective picture system; LF-rTMS = low-frequency repetitive transcranial magnetic stimulation; Likert = Likert scale; OCD = obsessive-compulsive disorder; ODT = objective distancing task; TPJ = temporoparietal junction; VAS = visual analogue scale; VLPFC = ventrolateral prefrontal cortex.

**Table 2:** Characteristics of included anodal transcranial direct current stimulation studies.

Reference	Design, Sample Type, Size *n*(Active)|*n*(Control)	Age (M ± SD or Range), Sex Ratio (M/F)	Electrode Positions (Localization Method)	Control Condition	Current Intensity, Size, Quantity	Protocol	Experiment Stimuli	Task (Emotion Regulation Strategy)	Outcome Measure
Wu et al. 2020 [53]	Within-subject, IGD, 33|~	21.21 ± 2.27, ~ ^1^	Right DLPFC (anode, F4; cathode, trapezius muscle)	Applied for only 60 s	1.5 mA, 5 × 7 cm^2^, 20 min	Online	Negative IAPS images	ERT (Reappraisal)	Perceived negative emotion in picture (Likert: 1–9)
Dam et al. 2021 [54] a	Between-subject, MDD, 11|8	24.11 ± 5.53, (2/17) ^2^	Left DLPFC (anode, F3; cathode, contralateral mastoid)	Applied for only 180 s	1.5 mA, 5 × 5 cm^2^, 20 min	Online	Negative IAPS images	ERT (Reappraisal)	Perceived negative emotion in picture (Likert: 1–4)
Dam et al. 2021 [54] b	Between-subject, Healthy, 11|7	23.94 ± 4.57, (6/12) ^2^	Left DLPFC (anode, F3; cathode, contralateral mastoid)	Applied for only 180 s	1.5 mA, 5 × 5 cm^2^, 20 min	Online	Negative IAPS images	ERT (Reappraisal)	Perceived negative emotion in picture (Likert: 1–4)
He et al. 2019 [50]	Between-subject, Healthy, 95|95	20.8 ± 2.3, (48/47) ^1^	Right VLPFC (anode, F6; cathode, FP1)	Applied for only 30 s	2.5 mA, 5 × 5 cm^2^, 34 min	Online	Social exclusion pictures	ERT (Reappraisal)	Perceived negative emotion in picture (Likert: 1–9)
He et al. 2018 [71] a	Between-subject, Healthy, 23|21	20.87 ± 1.4, (10/13) ^1^	Right VLPFC (anode, F6; cathode, FP1)	Applied for only 30 s	2.5 mA, 5 × 5 cm^2^, 24 min	Online	Social exclusion pictures	ERT (Reappraisal)	Perceived negative emotion in picture (Likert: 1–9)
He et al. 2018 [71] b	Between-subject, Healthy, 20|20	21.5 ± 1.4, (9/11) ^1^	Right VLPFC (anode, F6; cathode, FP1)	Applied for only 30 s	2.5 mA, 5 × 5 cm^2^, 24 min	Online	Social exclusion pictures	ERT (Reappraisal)	Perceived negative emotion in picture (Likert: 1–9)
Feeser et al. 2014 [73]	Between-subject, Healthy, 23|25	29.8 ± 6.2, (23/25) ^2^	Right DLPFC (anode, F4; cathode, left supraorbital)	Applied for only 30 s	1.5 mA, 35 cm^2^, 20 min	Online	Negative IAPS images	CRT (Reappraisal)	Perceived negative emotion in picture (Likert: 1–9)
Clarke et al. 2021 [75]	Between-subject, Healthy, 37|36	23.17 ± 6.77, (25/54) ^2^	Left DLPFC (anode, F3; cathode, left superior trapezius)	Applied for only 60 s	2.0 mA, 4 × 6 cm^2^, 20 min	Online	Negative IAPS images	ERT (Reappraisal)	Negative emotional experience (Likert: 0–12)
Marques et al. 2018 [55] a	Between-subject, Healthy, 30|30	23.1 ± 0.6, ~ ^1^	Left DLPFC (anode, F3; cathode, F4)	Applied for only 30 s	1.5 mA, 16 cm^2^, 20 min	Online	Negative IAPS images	CRT (Reappraisal)	Negative emotional experience (Likert: 1–9)
Marques et al. 2018 [55] b	Between-subject, Healthy, 30|30	22.0 ± 0.6, ~ ^1^	Right DLPFC (anode, F4; cathode, F3)	Applied for only 30 s	1.5 mA, 16 cm^2^, 20 min	Online	Negative IAPS images	CRT (Reappraisal)	Negative emotional experience (Likert: 1–9)
Marques et al. 2018 [55] c	Between-subject, Healthy, 29|30	20.62 ± 0.6, ~ ^1^	Left VLPFC (anode, F7; cathode, F8)	Applied for only 30 s	1.5 mA, 16 cm^2^, 20 min	Online	Negative IAPS images	CRT (Reappraisal)	Negative emotional experience (Likert: 1–9)
Marques et al. 2018 [55] d	Between-subject, Healthy, 30|30	21.1 ± 0.59, ~ ^1^	Right VLPFC (anode, F8; cathode, F7)	Applied for only 30 s	1.5 mA, 16 cm^2^, 20 min	Online	Negative IAPS images	CRT (Reappraisal)	Negative emotional experience (Likert: 1–9)
Chrysikou et al. 2019 [76] a	Between-subject, MDD, 10|10	24.20 ± 6.31, (6/14) ^2^	Left DLPFC (anode, F3; cathode, F4)	Applied for only 120 s	1.5 mA, 5 × 5 cm^2^, 20 min	Online	Negative IAPS images	ERT (Reappraisal)	Perceived emotion in picture (Likert: 0–8)
Chrysikou et al. 2019 [76] b	Between-subject, Healthy, 10|10	24 ± 4.38, (6/14) ^2^	Left DLPFC (anode, F3; cathode, F4)	Applied for only 120 s	1.5 mA, 5 × 5 cm^2^, 20 min	Online	Negative IAPS images	ERT (Reappraisal)	Perceived emotion in picture (Likert: 0–8)
Fink et al. 2019 [72] a	Within-subject, Healthy, 29|~	22.55 ± 5.44, (21/8) ^2^	VC (anode, Oz; cathode, Cz)	Applied for only 40 s	1mA, 25 cm^2^, 20 min	Online	Disgust-inducing pictures	ERT (Reappraisal)	Distress (Likert: 1–7)
Fink et al. 2019 [72] b	Within-subject, Healthy, 29|~	22.38 ± 3.43, (21/8) ^2^	Left DLPFC (anode, F3; cathode, Fp2)	Applied for only 40 s	1 mA, 25 cm^2^, 20 min	Online	Disgust-inducing pictures	ERT (Reappraisal)	Distress (Likert: 1–7)
Clarke et al. 2020 [74]	Between-subject, Healthy, 59|57	21.95 ± 5.88, (18/41) ^1^	Left DLPFC (anode, F3; cathode, left superior trapezius)	Applied for only 60 s	2.0 mA, 4 × 6 cm^2^, 20 min	Online	Negative IAPS images	ERT (Reappraisal)	Negative emotional experience (Likert: 0–12)
Doerig et al. 2021 [77]	Between-subject, Healthy, 25|25	24.36 ± 1.47, (16/9) ^1^	Right DLPFC (anode, F4; cathode, Cz)	Applied for only 15 s	1.5 mA, 5 × 7 cm^2^, 30 min	Online	Negative emotional memory	RT (Reappraisal)	Negative emotional experience (Likert: 1–10)

^1^ The demographical characteristics of the active group; ^2^ The demographical characteristics of the active and control groups; ~ The included studies did not explicitly report sex ratios or equal numbers of active and control groups in the within-subject study; a–d: Different effect sizes from the same study; CRT = cognitive reappraisal task; DLPFC = dorsolateral prefrontal cortex; ERT = emotion regulation task; IAPS = international affective picture system; IGD = internet gaming disorder; Likert = Likert scale; MDD = major depressive disorder; VC = visual cortex; RT = reappraisal and control task; VLPFC = ventrolateral prefrontal cortex.

**Table 3:** Results of subgroup analyses for categorical moderators.

Variable	*k*	Hedges’ g (95% CI) ^a^	*z*	*p* ^b^	*Q*	*p* ^c^
Target electrode placement ***					49.79	0.000
lDLPFC	6	−0.11 (−0.19 to 0.42)	0.71	0.479		
rVLPFC *	4	−0.45 (−0.81 to −0.08)	−2.41	0.016		
rDLPFC **	3	−0.69 (−1.11 to −0.27)	−3.24	0.001		
lVLPFC ***	1	−2.42 (−3.21 to −1.69)	−6.31	0.000		
VC	1	−0.02 (−0.67 to 0.64)	−0.05	0.959		
Return electrode placement *					11.84	0.019
Supraorbital *	5	−0.60 (−1.08 to −0.12)	−2.43	0.015		
Opposite brain area (bilateral tDCS)	5	−0.55 (−1.29 to 0.19)	−1.45	0.147		
Extracephalic ^•^	3	0.55 (−0.07 to 1.16)	1.75	0.080		
Vertex ^•^	2	−0.57 (−1.19 to 0.06)	−1.78	0.074		

^a^ According to the random-effects model; ^b^ The *p* value indicates whether the difference between the effect sizes in the subgroups is significant; ^c^ The *p* value of the *Q*-test for moderation; lDLPFC = left dorsolateral prefrontal cortex; lVLPFC = left ventrolateral prefrontal cortex; rDLPFC = right dorsolateral prefrontal cortex; rVLPFC = right ventrolateral prefrontal cortex; VC = visual cortex; ^•^ *p* < 0.1; * *p* < 0.05; ** *p* < 0.01; *** *p* < 0.001.

**Table 4:** Results of meta-regression analyses for continuous moderators.

Variable	*β*	SE	LL	UL	*z*	*p* ^a^	*Q*	*p* ^b^
Current intensity							0.00	0.991
Intercept	−0.26	0.93	−2.08	1.56	−0.28	0.783		
Slope	0.00	0.51	−0.99	1.00	0.01	0.991		
Target electrode size							1.36	0.243
Intercept	0.94	1.04	−1.10	2.97	0.90	0.367		
Slope	−0.05	0.04	−0.13	0.03	−1.16	0.243		
Duration of intervention							0.57	0.451
Intercept	0.56	1.10	−1.59	2.72	0.51	0.609		
Slope	−0.04	0.04	−0.13	0.06	−0.75	0.451		

^a^ The *p* value indicates whether the difference between the effect sizes in the subgroups are significant; ^b^ The *p* value of the *Q*-test for moderation; SE = standard error of the coefficient; LL = lower limit of the 95% CI; UL = upper limit of the 95% CI.

## Data Availability

Not applicable.

## Supplementary Materials

The following supporting information can be downloaded at: https://www.mdpi.com/2076-3425/12/6/786/s1, Table S1: PEDro scores assigned to all included studies.
