# Correction: Zhang et al. The Effect of Non-Invasive Brain Stimulation on the Downregulation of Negative Emotions: A Meta-Analysis. *Brain Sci*. 2022, *12*, 786

**DOI:** 10.3390/brainsci12081107

**Published:** 2022-08-19

**Authors:** Qingqing Zhang, Xiaoming Li, Xinying Liu, Shanshan Liu, Mengzhu Zhang, Yueling Liu, Chunyan Zhu, Kai Wang

**Affiliations:** 1 The School of Mental Health and Psychological Sciences, Anhui Medical University, Hefei 230032, China; 2 Anhui Province Key Laboratory of Cognition and Neuropsychiatric Disorders, Hefei 230032, China; 3 Collaborative Innovation Center of Neuropsychiatric Disorders and Mental Health, Hefei 230032, China; 4 Institute of Artificial Intelligence, Hefei Comprehensive National Science Center, Hefei 230011, China; 5 Department of Neurology, The First Affiliated Hospital of Anhui Medical University, Hefei 230022, China

## 1. Error in Figure

In the original article [1], there was a mistake in original Figure 1. PRISMA flow diagram of literature search and study selection as published. There are incorrect *n* values in original Figure 1. The corrected Figure 1 appears below. 

In the original article, there was a mistake in orignal Figure 4. Results of the meta-analyses for tDCS studies as published. In orignal Figure 4A, the last line of 95%CI, “[−0.69, 0.17]” should be “[−0.69, 0.19]”. The corrected Figure 4 appears below. 

## 2. Text Correction

There was an error in the original article. In Paragraph 1, Section 3.3.2. rTMS and Section 4. Discussion, “g = −0.42” should be corrected to “g = −0.43”. The corrected text is as below:

In Paragraph 1, Section 3.3.2. rTMS:

We separately analyzed the data of four studies in the downregulating condition and the maintaining condition, including 185 healthy participants. Of these, 109 only received active stimulation, and 76 only received sham stimulation. The results of the analysis in downregulating condition showed no significant heterogeneity (*Q* = 8.79, *p* = 0.118, I^2^ = 43.15%) and that self-reported emotion significantly differed between active and sham stimulation groups (g = −0.43, CI_95%_ = [−0.85, −0.00], Z-value = −1.98, *p* = 0.048, Figure 3A). The results of the analysis in maintaining condition also showed no significant heterogeneity (*Q* = 5.97, *p* = 0.113, I^2^ = 35.05%), but we found no significant difference between active and sham stimulation (g = −0.24, CI_95%_ = [−0.73, 0.25], Z-value = −0.96, *p* = 0.335, Figure 3B). 

In Paragraph 1, Section 4. Discussion:

This meta-analysis investigated the effects of NIBS on the downregulation of negative emotions and the maintenance of negative emotions in single-session designs with healthy or clinical populations. Overall, we documented a small and significant excitatory effect of NIBS on the downregulation of negative emotions (g = −0.29), and a significant effect on the maintenance of negative emotions (g = −0.19). Further analyses showed that rTMS had a medium and significant effect on the downregulation of negative emotions in healthy populations (g = −0.43), but had no significant effect on the maintenance of negative emotions, while tDCS also had no significant effect on the downregulation and maintenance of negative emotions in healthy populations. 

The authors state that the scientific conclusions are unaffected. This correction was approved by the Academic Editor. The original publication has also been updated.

## Figures and Tables

**Figure 1:**
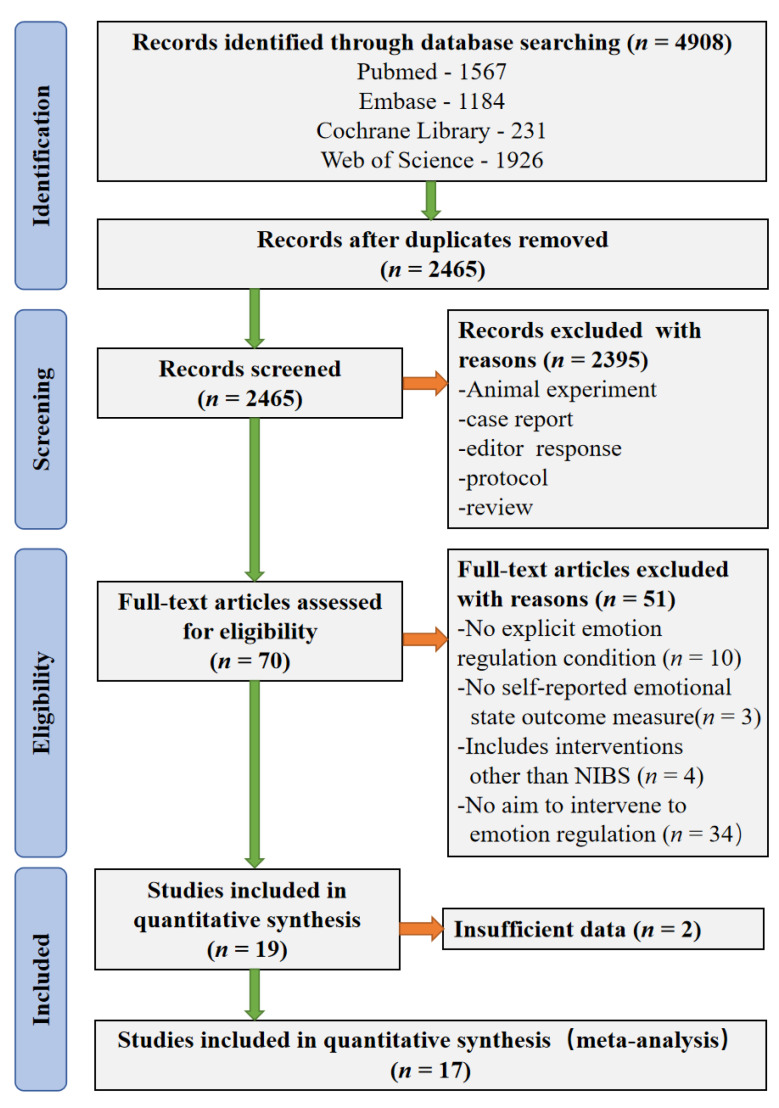
PRISMA flow diagram of literature search and study selection.

**Figure 4:**
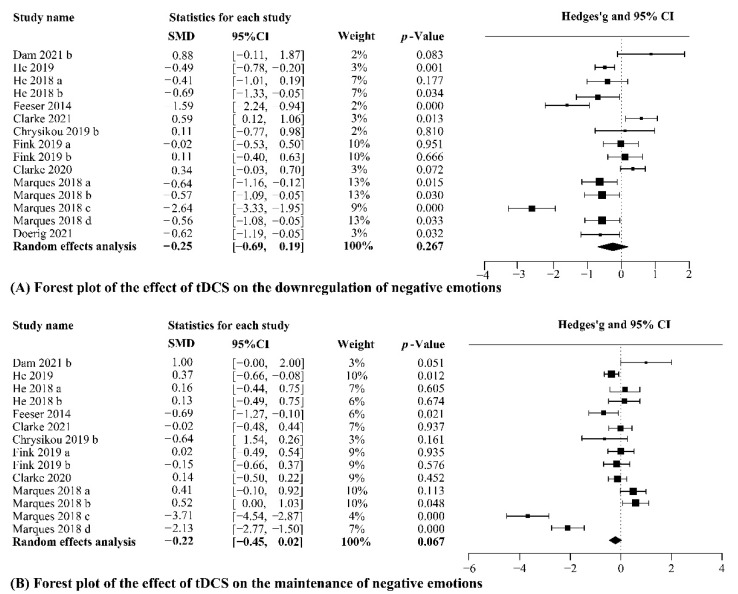
Results of the meta-analyses for tDCS studies. (a–d) Different effect sizes from the same study [50,54,55,71,72,73,74,75,76,77].
