# Supplementary material for: The Effect of Non-Invasive Brain Stimulation on the Downregulation of Negative Emotions: A Meta-Analysis
Source: Brain Sci. 2022 Jun 15;12(6):786. doi: 10.3390/brainsci12060786 (PMC9221395; doi:10.3390/brainsci12060786)
Supplement: Supplementary file 1 [file brainsci-12-00786-s001.zip › brainsci-1738345-supplementary.pdf]

Supplementary Material

Part1. Elaborate description of the search term

PubMed:

((((((((emotion regulation[Title/Abstract])) OR (reappraisal[Title/Abstract])) OR (suppression[Title/Abstract])) OR (distraction[Title/Abstract])) OR (rumination[Title/Abstract])) OR (acceptance[Title/Abstract])) OR (avoidance[Title/Abstract])) AND (((((noninvasive brain stimulation[Title/Abstract])) OR (transcranial direct current stimulation[Title/Abstract])) OR (transcranial magnetic stimulation[Title/Abstract])) OR (theta burst stimulation[Title/Abstract])) OR (transcranial electrical stimulation[Title/Abstract])

Web of science：

TS=(noninvasive brain stimulation* OR transcranial direct current stimulation* OR transcranial magnetic stimulation* OR theta burst stimulation* OR transcranial electrical stimulation) AND
TS=(emotion regulation* OR reappraisal* OR suppression* OR distraction* OR rumination* OR acceptance* OR avoidance)

Cochrane library:

#1=(noninvasive brain stimulation):ti,ab,kw OR (transcranial direct current stimulation):ti,ab,kw OR (transcranial magnetic stimulation):ti,ab,kw OR (theta burst stimulation):ti,ab,kw OR (transcranial electrical stimulation):ti,ab,kw (Word variations have been searched)
#2=(emotion regulation):ti,ab,kw OR (reappraisal):ti,ab,kw OR (suppression):ti,ab,kw OR (distraction):ti,ab,kw OR (rumination):ti,ab,kw
#3=#1 AND #2

Embase:

('noninvasive brain stimulation':ab,ti OR 'transcranial direct current stimulation':ab,ti OR 'transcranial magnetic stimulation':ab,ti OR 'theta burst stimulation':ab,ti OR 'transcranial electrical stimulation':ab,ti) AND
('emotion regulation':ab,ti OR reappraisal:ab,ti OR suppression:ab,ti OR distraction:ab,ti OR rumination:ab,ti OR acceptance:ab,ti OR avoidance:ab,ti)

Part 2: **SUPPLEMENTARY TABLES**

Table S1. PEDro scores assigned to all included studies

	Q1	Q2	Q3	Q4	Q5	Q6	Q7	Q8	Q9	Q10	Q11	Total
**Zhao (2021)**	1	1	0	1	1	0	1	1	1	1	1	8
**He (2020)**	1	1	0	1	1	0	1	1	1	1	1	8
**De Wit (2015)**	1	0	0	1	1	0	1	1	1	1	1	7
**Cao (2021)**	1	0	0	1	1	0	1	1	1	1	1	7
**Jansen (2019)**	1	0	0	1	1	0	1	1	1	1	1	7
**Wu (2020)**	1	1	0	1	1	1	1	1	1	1	1	9
**Van Dam (2021)**	0	0	0	1	1	0	1	1	1	1	1	7
**He (2020)**	1	1	0	1	1	0	1	1	1	1	1	8
**He (2018)**	1	1	0	1	1	0	1	1	1	1	1	8
**Feeser (2014)**	1	1	0	1	1	1	1	1	1	1	1	9
**Clarke (2021)**	1	0	0	1	1	0	1	1	1	1	1	7
**Chrysikou (2019)**	1	1	0	1	1	0	1	1	1	1	1	8
**Fink (2019)**	1	0	0	1	1	0	1	1	1	1	1	7
**Clarke (2020)**	1	0	0	1	1	0	1	1	1	1	1	7
**Marques (2018)**	1	0	0	1	1	0	1	1	1	1	1	7
**Powers (2020)**	1	1	0	1	1	1	1	1	1	1	1	9
**Doerig (2021)**	1	1	1	1	1	1	1	1	1	1	1	11

Q1: Eligibility criteria were specified

Q2: Subjects were randomly allocated to groups (in a crossover study, subjects were randomly allocated an order in which treatments were received)

Q3: Allocation was concealed

Q4: The groups were similar at baseline regarding the most important prognostic indicators

Q5: There was blinding of all subjects

Q6: There was blinding of all therapists who administered the therapy

Q7: There was blinding of all assessors who measured at least one key outcome

Q8: Measures of at least one key outcome were obtained from more than 85% of the subjects initially allocated to groups

Q9: All subjects for whom outcome measures were available received the treatment or control condition as allocated or,where this was not the case, data for at least one key outcome was analyzed by "intention to treat"

Q10: The results of between statistical comparisons are reported for at least one key outcome

Q11: The study provides both point measurements and measurements of variability for at least one key outcome
